# Effectiveness and therapeutic compliance of digital therapy in shoulder rehabilitation: a randomized controlled trial

**DOI:** 10.1186/s12984-023-01188-7

**Published:** 2023-07-08

**Authors:** Alex Rizzato, Martina Pizzichemi, Erica Gobbi, Adriana Gerardi, Claudia Fortin, Ancuta Copcia, Antonio Paoli, Giuseppe Marcolin

**Affiliations:** 1 Department of Biomedical Sciences, University of Padova, Via Marzolo, 3, Padova, 35131 Italy; 2 School of Human Movement Sciences, University of Padova, Padova, Italy; 3 Department of Biomolecular Sciences, University of Urbino Carlo Bo, Urbino, Italy; 4 Data Medica group, Synlab S.p.A, CEMES, Padova, Italy

**Keywords:** Digital therapy, Shoulder rehabilitation, Engagement, Pain, Strength, Range of motion

## Abstract

**Background:**

Interactive videogames, virtual reality, and robotics represent a new opportunity for multimodal treatments in many rehabilitation contexts. However, several commercial videogames are designed for leisure and are not oriented toward definite rehabilitation goals. Among the many, Playball^®^ (Playwork, Alon 10, Ness Ziona, Israel) is a therapeutic ball that measures both movement and pressure applied on it while performing rehabilitation games. This study aimed: (i) to evaluate whether the use of this novel digital therapy gaming system was clinically effective during shoulder rehabilitation; (ii) to understand whether this gaming rehabilitation program was effective in improving patients’ engagement (perceived enjoyment and self-efficacy during therapy; attitude and intention to train at home) in comparison to a control non-gaming rehabilitation program.

**Methods:**

A randomized controlled experimental design was outlined. Twenty-two adults with shoulder pathologies were recruited for a rehabilitation program of ten consecutive sessions. A control (CTRL; N = 11; age: 62.0 ± 10.9 yrs) and an intervention group (PG; N = 11; age: 59.9 ± 10.2 yrs) followed a non-digital and a digital therapy, respectively. The day before (T_0_) and after (T_1_) the rehabilitation program, pain, strength, and mobility assessments were performed, together with six questionnaires: PENN shoulder Score, PACES-short, Self-efficacy, Attitudes to train at home, Intention to train at home, and System usability scale (SUS).

**Results:**

MANOVA analysis showed significant improvements in pain (p < 0.01), strength (p < 0.05), and PENN Shoulder Score (p < 0.001) in both groups. Similarly, patients’ engagement improved, with significant increments in Self-efficacy (p < 0.05) and attitude (p < 0.05) scores in both groups after the rehabilitation. Pearson correlation showed significant correlations of the Δ scores (T_1_ - T_0_) between PACES and Self-efficacy (r = 0.623; p = 0.041) and between PACES and Intention to train at home (r = 0.674; p = 0.023) only in the PG. SUS score after the rehabilitation (74.54 ± 15.60) overcame the cut-off value of 68, representative of good usability of a device.

**Conclusions:**

The investigated digital therapy resulted as effective as an equivalent non-digital therapy in shoulder rehabilitation. The reported positive relationship between the subject’s enjoyment during digital therapy and intention to train at home suggests promising results in possible patient’s exercise engagement at home after the rehabilitation in the medical center.

**Retrospectively registered:**

NCT 05230056.

## Introduction

The latest development in technologies and connectivity, together with ever-growing computing, networking, and sensing power, is progressively changing people’s habits [1]. Nowadays, the employment of digital equipment and devices represents a focal core in most areas of society, including health and medical monitoring. Digital therapy with interactive games, virtual reality, websites, and robotics represents a new opportunity for multimodal treatments in many rehabilitation contexts [2]. About that, the Nintendo Wii has been regularly used as a rehabilitation tool in 61% of Australian rehabilitation centers to treat people post-stroke [3]. Indeed, visual and sound feedbacks are essential to improve motor control in pathologies or injury treatment and prevention [4, 5]. Low-cost devices such as the Kinect sensor or the Nintendo Wii Balance Board have been reported as playful and motivating tools in rehabilitating children with cerebral palsy [6]. In the pediatric field, De Kloet and colleagues reported that children with acquired brain injury had cognitive and motor benefits following a 12-week tailored rehabilitation program with the Nintendo Wii [7]. The rationale behind the increased application of digital therapies is to improve patients’ motivation influencing their affective response and engagement [8, 9]. Additionally, since subjects can perceive therapeutic exercises as monotonous, an interactive approach has been demonstrated to improve the compliance of patients [9]. Moreover, after completing the rehabilitation, virtual reality or gaming interventions showed improved treatment adherence, engagement [10], and quality of life over conventional treatment of upper limb functions in patients with stroke [11]. However, many commercial video games are designed for leisure and are not oriented toward definite rehabilitation goals. Noteworthy, motivational factors related to engagement in rehabilitation though gaming systems are still limited [12]. Although no specific recommendations exist on video games as a rehabilitation tool, several studies have shown promising results on specific parameters of upper limb function, gross motor function, and pain reduction [13–15]. In this context, the device PlayBall^®^ (Playwork, Alon 10, Ness Ziona, Israel) is a novel digital therapy gaming system with possible motivational assets in physiotherapy. Specifically, PlayBall^®^ is a smart exercise ball functioning as a performance-measuring tool and videogame controller that allows patients to complete rehabilitation games and receive real-time visual feedback. The interactive ball allows measuring both movement and pressure applied on it. Smart integrated sensors objectively measure and track the performance to monitor the patient’s progress. Therefore, the objectives of this study were: (i) to evaluate whether the use of a novel digital therapy gaming system (PlayBall^®^) was clinically effective during shoulder rehabilitation; (ii) to understand whether the gaming rehabilitation program was effective in improving patients’ engagement in comparison to a control non-gaming rehabilitation program. Particularly, enjoyment and self-efficacy during therapy, together with attitude and intention to train at home after the rehabilitation, were assumed as measures of the patients’ engagement [10].

## Methods

### Subjects

Subjects were recruited from a medical center (CEMES, Data Medica group, Synlab S.p.A., Padova, Italy). The following inclusion and exclusion criteria were considered for recruitment. Inclusion criteria: (i) presence of one of the following shoulder pathologies: impingement syndrome, capsulitis, tendon injuries, degenerative joint or tendon pathologies; (ii) pain between 2/10 and 8/10 on a visual analogue scale. Exclusion criteria: (i) post-surgical patients; (ii) inability to perform active exercises; (iii) peripheral neurological deficits; (iv) cervical-brachialgia; (v) algodystrophy. The choice of the sample size was based on an a priori power analysis (G*Power Version 3.1.9.4). Based on the MANOVA, we obtained a sample size of 22 subjects considering as input a large effect size (f = 0.40), p = 0.05, and Power (1-β error probability) = 0.7. Twenty-eight subjects were screened, but six did not accept to participate in the study. Thus, twenty-two subjects (age = 61 ± 10.4 years; F = 16; M = 6) were finally enrolled.

### Experimental design

The experimental protocol received approval by the Human Ethical Committee of the Department of Biomedical Sciences of the University of Padova (n° HEC-DSB/02–21; NCT 05230056) and adhered to the principles of the Declaration of Helsinki. All the subjects, informed about the methods of the study, gave their written informed consent and were free to renounce the study at any stage. Specifically, they were informed that, before and after their shoulder rehabilitation protocols, they should have performed a strength and mobility assessment, and filled in some questionnaires. Subjects were randomly divided (https://www.graphpad.com/quickcalcs/randomize1/) by a researcher of the Department of Biomedical Sciences not involved in the study and unaware of the aims of the research as follows: a group who underwent digital therapy with the Playball^®^ device (PG; N = 11; age: 59.9 ± 10.2 yrs) and a group who followed an equivalent but non-digital rehabilitation program (CTRL; N = 11; age: 62.0 ± 10.9 yrs). All participants were blinded with respect to the differences between the two therapeutic protocols.

We outlined a randomized controlled trial in which both CTRL and PG underwent ten consecutive shoulder rehabilitation sessions (from Monday to Friday, excluding Saturday and Sunday). In particular, the daily rehabilitation session lasted 40 min. Within each session, the first 20 min, the subjects were treated manually by the physiotherapist, while for the last 20 min, they performed active exercises to improve shoulder strength and mobility. Physiotherapists performed an equal manual treatment for both CTRL and PG. Conversely, active exercises supervised by researchers had the same goals but differed in the exercising modality that was non-digital for CTRL and digital for PG. The day before and after the rehabilitation period, subjects participated in an evaluation session comprising functional and self-reported measures. The shoulder rehabilitation of all the patients were administered in the same medical center (CEMES, Data Medica group, Synlab S.p.A., Padova, Italy).

### Functional assessments

The shoulder mobility and strength assessments were carried out the day before (T_0_) and the day after (T_1_) the rehabilitation period by physiotherapists blind to the group allocation.

#### Mobility assessment

Shoulder mobility was measured with a wireless inertial sensor (Gyko, Microgate Italia - Bolzano, Italy) at a sampling frequency of 500 Hz. Following the manufacturer’s guidelines, the Gyko was applied to the injured arm with an elastic band at the distal level of the humerus, right above the elbow joint. The mobility assessment was performed for arm flexion, abduction, external rotation, and extension. Subjects performed all movements while seated with their backs on the wall, excluding the extension movement that was performed while standing. Subjects were instructed to perform all the movements at a self-selected speed and range of motion (ROM) with the indication not exceeding shoulder pain limits. Overall, three trials were performed, with a recovery of 30 s in between. Data were analyzed with the software GykoRePower (Gyko, Microgate Italia - Bolzano, Italy) to obtain the ROM (deg) and mean velocity (deg/s) of each movement.

#### Strength assessment

The Playball® was employed to measure the maximal isometric strength (F_max_) of the injured shoulder. The device was positioned under the subject’s hand and over a solid surface. From a seated position, the subject was asked to push down the ball as strongly as possible with the shoulder abducted to 70 deg and elbow extended. Three maximal voluntary contractions (MVC) lasting three seconds each, with a between-trial recovery of 30 s, were performed. During the MVC test, the subject had real-time visual feedback of the force on a tablet.

### Questionnaires

A questionnaire package was used to assess three different dimensions: (a) the shoulder global health status; (b) the engagement response; (c) the usability of the system. In all cases, the questionnaires were administered in a quiet room. The researcher made clear what each dimension of the questionnaire meant and clarified any queries. The subjects were not asked to give their names and were ensured that answers would remain anonymous. Questionnaires were administered on the same days the shoulder mobility and strength assessment occurred. A questionnaire for the usability of the system was administered only to PG. Moreover, during the mobility assessment, the level of pain corresponding to the execution of each movement was assessed with a visual analogue scale (VAS) [16]. The pain levels assessed after each movement (i.e., flexion, abduction, external rotation, and extension) with VASs were averaged in a single score as output (VAS-PAIN).

#### Shoulder global health status

The PENN Shoulder Score was employed to investigate the shoulder global health status. It consists of three sections that measure shoulder pain, satisfaction, and function. The pain section quantifies the pain of the shoulder at rest, during common and strenuous activities, for a maximum score of 30 points, indicating the absence of pain. The shoulder satisfaction corresponds to a single item for a maximum score of 10 points representing the highest degree of satisfaction. The functionality section consists of 20 items on a four-point Likert scale: 0 points (impossible to perform), 1 point (very difficult), 2 points (some difficult), and 3 points (no difficult). The subjects were asked to focus on the injured shoulder while performing daily living activities. The three-section best score is 100 points, indicating the absence of pain, high functionality, and good subject’s satisfaction of the shoulder [17].

#### Enjoyment

An adapted version of the Italian Physical Activity Enjoyment Scale (PACES) [18] was used to assess pleasure during therapy. The PACES discriminates between pleasant and unpleasant experiences associated with physical activity. For this study, the PACES 12-item version [19] was used adapting the stem in “When I perform the therapeutic exercises, I …”, and participants rated their agreement with 6 positive (e.g., “*I enjoy*”) and 6 negative (e.g., “*I feel bored*”) items on five-point Likert scale (1 = “*totally disagree*” to 5 = “*completely agree*”). The total score ranged from 12 to 60 and Cronbach’s α was 0.82 for T_0_ and 0.92 for T_1_.

#### Self-efficacy

Self-efficacy is defined as the perceived ability to plan and execute specific behaviors [20]; it is associated with the intention to perform the specific behavior. Self-efficacy in performing exercises during intervention was studied with a one-item scale (i.e., “How confident are you in your ability to perform the therapeutic exercises correctly?”) ranging from 1 (*not confident at all*) to 7 (*absolutely confident*).

#### Attitude to train at home

Attitudes towards a behavior (i.e., a positive or negative predisposition towards a specific behavior) are crucial in the intention to perform that behavior [21]. Precisely, attitudes towards exercise at home were estimated by the mean score of responses to the question “*Doing shoulder exercises one hour a day at home after the rehabilitation cycle is…*”. Responses were rated on a 7-point Likert-type scale on six bipolar adjectives: bad/good, wrong/right, unpleasant/pleasant, useless/useful, difficult/easy, and boring/funny.

#### Intention to train at home

Intention can be defined as the will to perform a specific behavior and it is considered the most proximal antecedent to the behavior itself. Considering the theory of planned behavior [21], the intention to exercise at home was assessed using a two-item questionnaire [22]. A seven-point scale ranging from 1 (“*totally disagree*”) to 7 (“*totally agree*”) was used for each item. Answers were given to the following statements: (i) *“after the therapy period, I intend to perform shoulder exercises twenty minutes per three times a week at home”*; (ii) *“after the therapy period, I am determined to perform shoulder exercises twenty minutes per three times a week at home”*. The scores were averaged to compute a mean score.

#### Usability

The Italian version of the System Usability Scale (SUS), [23] was employed to assess the Playball® system. The SUS is a ten-item questionnaire that operationally defines the subjective perception of interaction with a system [24]. Items from SUS considered the following sections: (i) subjects’ ability to complete activities using the system and the quality of the output of activities performed (i.e., effectiveness); (ii) the level of resources consumed in carrying out the tasks (i.e., efficiency); (iii) the individual feelings and reactions of subjects using the system (i.e., satisfaction). Moreover, seven VASs (from 0 to 10) were employed to investigate the following dimensions: contact with the tool, controls, perception of security, general comfort, readability of the data, aesthetic pleasantness, and general pleasure; then generating a global averaged score named VAS-US measure.

### Therapeutic protocol

The exercise intervention for both CTRL and PG consisted of a progressive strength and mobility program to be performed in the medical center with the supervision of the physiotherapists, not associated with either study condition.

**Fig. 1:**
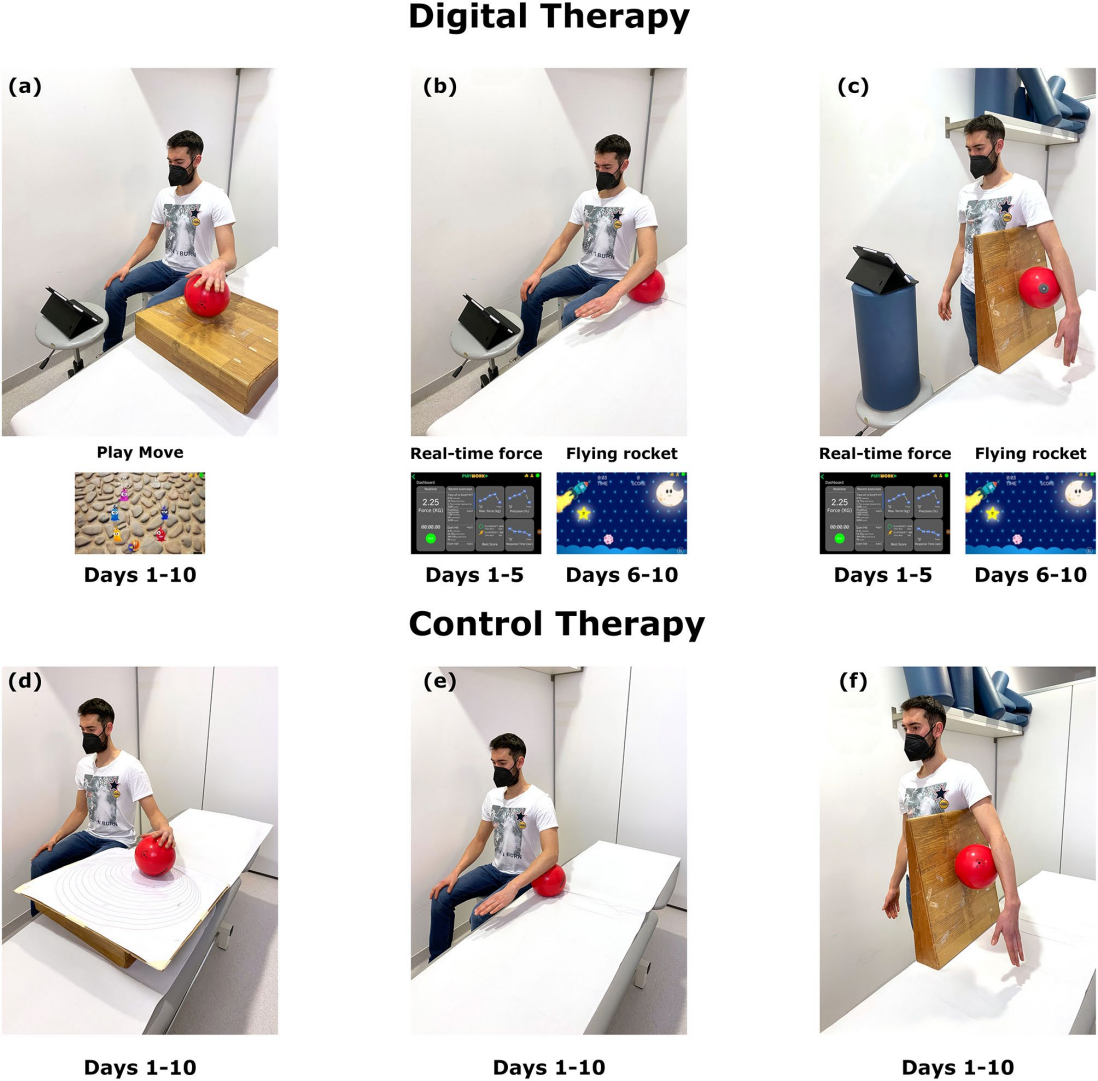
Graphical summary of the rehabilitation protocol. Digital therapy is represented in the upper panels together with the screenshots of the gaming interface: “PlayMove” exercise performed from the seated position on a 30-deg inclined surface (**a)**; “Real-time force” and “Flying rocket” exercise from the seated (**b)** and standing **(c)** positions. Control therapy is represented in the lower panels: Perfetti’s circles exercise performed on a 30-deg inclined board **(d)**; isometric contractions from the seated **(e)** and standing (**f**) positions

**Digital therapy.** The digital therapy allowed the subject to complete the rehabilitation program while playing games and receiving real-time visual feedback. A single digital therapy session was organized in three different 4-minute bouts, including the “PlayMove” exercise and the “Real-time force” exercise from seated and standing positions. The “PlayMove” exercise was performed on a 30-deg inclined surface (Fig. 1a): the exercise started guiding the PlayBall^®^ with the hand until session 3 and then with the elbow. After the fourth session, a contextual pressure to the circular motion of the cursor was added for two sessions at 2%, 5%, and 10% of the measured F_max_, respectively. In the “Real-time force” exercise from the seated position (Fig. 1b), the subject was required to perform subsequent isometric contractions on the Playball^®^ with the elbow flexed at 90 deg. The tablet, positioned in front of the subject, allowed real-time feedback on the force applied. Particularly, the patient was asked to push down and release the Playball^®^ as follows: from session one, 3s contraction and 3s recovery; from session three, 4s contraction and 2s recovery; in session five, 5s contraction and 2s recovery. Patients performed 3 sets of 10 repetitions. “Real-time force” exercise was also performed from the standing position with the elbow extended. The Playball^®^ was placed on a wedge and under the subject’s arm (Fig. 1c**)**. From sessions one to five, the repetitions were the same as for the seated position. From session six, a new strength exercise, the “Flying rocket”, substituted both the seated and standing “Real-time force” exercise. In this new exercise, the subject was required to guide a spaceship, pushing down and releasing the Playball^®^, aiming to hit stars and avoid asteroids.

**Control therapy.** Subjects completed a rehabilitation program during the control therapy without interactive processes or visual feedback. As per the digital therapy, the single session was organized in three different 4-minute bouts, including Perfetti’s circles and isometric contractions from seated and standing positions. In Perfetti’s circles (Fig. 1d), the subject had to move a ball with the hand following specific circumferences drawn on a 30-deg inclined board. Specifically, the board had 20 concentric circles, tangents in one point, and an increasing radius. The exercise was performed clockwise and counterclockwise. The isometric contractions from seated (Fig. 1e) and standing (Fig. 1f) positions were equal to those performed by the PG but without visual feedback on the amount of force applied to the ball.

### Statistical analysis

The mean value and the standard deviations (SD) were calculated for each variable. The D’Agostino-Pearson test was employed to check the data normality distribution. An unpaired sample T-test was used for baseline (T_0_) comparisons between groups (CTRL vs. PG) on demographic data, functional variables, and shoulder global health status. Moreover, a paired-T-test was used to compare PRE (T_0_) and POST (T_1_) results of SUS and VAS-US in the PG. A multivariate analysis of variance (MANOVA) was used to compare functional and engagement variables between the two time points (T_0_ vs. T_1_) and the two groups (CTRL vs. PG). Finally, for both CTRL and PG, the Pearson correlation analysis was performed to investigate the relationship between engagement variables considering the differences between T_0_ and T_1_ (Δ scores). The significance level was set at p < 0.05. All analyses were performed using Statistical Package for Social Sciences (SPSS) version 27 (IBM, Armonk, New York, USA).

## Results

All of the subjects completed the study. Unpaired T-test for baseline comparisons (T_0_) showed no statistically significant differences between CTRL and PG for demographic data, functional variables, and shoulder global health status (Table 1). The MANOVA analysis revealed a significant main effect of time (T_0_ vs. T_1_) for F_max_ (p < 0.05), VAS-PAIN values (p < 0.01), and the PENN Shoulder Score (p < 0.001), (Table 2). Conversely, no significant main effect of groups (CTRL vs. PG) was observed.

**Table 1:** Baseline comparisons for all the variables. Data are expressed as mean ± standard deviation (SD). ns: not statistically significant.

	CTRL (T_0_)	PG (T_0_)	p value
Age (yrs)	62.00 ± 10.20	59.91 ± 10.93	ns
BMI (kg/m^2^)	25.81 ± 4.24	26.39 ± 4.21	ns
F_max_ (kg)	15.16 ± 6.72	12.20 ± 5.94	ns
ROM (deg)	309.30 ± 62.66	286.38 ± 57.30	ns
VELOCITY (deg/s)	42.83 ± 26.44	39.05 ± 20.14	ns
VAS-PAIN	4.30 ± 2.52	4.04 ± 2.19	ns
PENN shoulder score	54.70 ± 14.55	54.30 ± 12.64	ns

**Table 2:** Results of the MANOVA analysis for the functional variables (i.e., F_max_, ROM, and VELOCITY), the VAS-PAIN, and the PENN Shoulder Score. Post-hoc comparisons show the significant main effect of time (T_0_ vs. T_1_) for both control (CTRL) and PlayBall (PG) group. Significantly different from PRE (T_0_): * (p < 0.05); ** (p < 0.01); *** (p < 0.001)

	CTRL		PG	
	PRE (T_0_)	POST (T_1_)	PRE (T_0_)	POST (T_1_)
F_max_ (kg)	15.16 ± 6.71	18.85 ± 9.16 *	12.20 ± 5.94	15.35 ± 10.67 *
ROM (deg)	309.30 ± 62.65	325.36 ± 70.20	286.38 ± 57.29	283.97 ± 60.47
VELOCITY (deg/s)	42.83 ± 26.44	47.48 ± 24.50	39.05 ± 20.14	40.69 ± 14.84
VAS-PAIN	4.30 ± 2.52	2.70 ± 2.02 **	4.04 ± 2.19	3.17 ± 2.49 **
PENN shoulder score	54.70 ± 14.55	67.29 ± 16.41 ***	54.30 ± 12.64	63.84 ± 13.96 ***

Moreover, MANOVA analysis showed a significant main effect of time (T_0_ vs. T_1_) only for scores from self-efficacy (p < 0.05) and attitude to train at home (p < 0.05); post-hoc comparisons are presented in Table 3. Again, no significant main effect of groups (CTRL vs. PG) was observed in any of the engagement variables.

**Table 3:** Results of the MANOVA analysis for the engagement variables. Post-hoc comparisons show the significant main effect of time (T_0_ vs. T_1_) for both control (CTRL) and PlayBall (PG) groups. Significantly different from PRE (T_0_): * (p < 0.05)

	CTRL		PG	
	PRE (T_0_)	POST (T_1_)	PRE (T_0_)	POST (T_1_)
PACES	53.90 ± 4.52	51.63 ± 10.97	50.18 ± 9.44	51.63 ± 8.41
Self-efficacy	5.36 ± 1.20	6.18 ± 0.87 *	5.0 0 ± 1.09	5.18 ± 1.07 *
Attitude to train at home	5.67 ± 1.59	6.29 ± 0.66 *	4.67 ± 1.24	5.48 ± 1.41 *
Intention to train at home	6.09 ± 1.62	5.90 ± 1.85	5.50 ± 1.56	6.18 ± 1.07

Exclusively in the PG, paired T-test did not show any significant differences between PRE vs. POST comparisons considering the score from SUS (T_0_: 67.72 ± 9.96; T_1_: 74.54 ± 15.60) and the mean score from VAS-US (T_0_: 7.78 ± 1.62; T_1_: 7.97 ± 1.53).

Finally, the Pearson correlation analysis showed statistically significant correlations between the Δ scores (T_1_ - T_0_) of engagement variables in the PG. Positive significant correlations were found between Δ scores from “PACES” and “Self-efficacy” (r = 0.623; p = 0.041) and between Δ scores from “PACES” and “Intention to train at home” (r = 0.674; p = 0.023). In the CTRL, no significant correlations were found considering the Δ scores of engagement variables.

## Discussion

The present study aimed to determine the effectiveness of a digital therapy performed with the Playball® device during a shoulder rehabilitation protocol. Both the therapeutic and engagement responses to the digital therapy were investigated and compared to a non-digital equivalent rehabilitation protocol. The main finding was that the rehabilitation carried out with the Playball® was as effective as the equivalent non-digital rehabilitation. Namely, both proposed rehabilitation programs effectively improved shoulder recovery, improved strength, reduced pain, and globally increased the subjects’ self-perceived satisfaction and functionality.

Our findings align with previous research [25–27] and revealed that digital therapy could also be a viable therapeutic modality in shoulder rehabilitation. Specifically, the digital therapy effectively reduced pain throughout the range of motion and regained strength levels in short-term shoulder rehabilitation. Our results agree with a pilot study on shoulder impingement where a Nintendo Wii-based protocol reduced pain and disability, improving quality of life and pain-free shoulder ROM in the sagittal and frontal planes [27]. The effectiveness of digital therapy in shoulder rehabilitation adds to that already reported in hemiplegic post-stroke patients [26] as well in patients with Parkinson’s disease [25].

Concerning the engagement variables, the scores from Self-efficacy and Attitude to train at home dimensions significantly improved following the shoulder rehabilitation, regardless of therapy. Therefore, the repetition of therapeutic exercises and the shoulder functional improvements could have influenced the higher subjects’ self-efficacy and attitude to train at home. Thus, the longer the rehabilitation time, the more the subjects feel self-confident, and consequently, their will to exercise at home increases. It is well accepted that repeated practice, such as during an exercise training period, increases the subject’s confidence and motor learning [28–30]. In our case, the rehabilitation sessions allowed the subjects to become more familiar with the exercises over time, thus, improving the perception of their ability and performance. Self-efficacy, firstly stated by Bandura in the social cognitive theory [31], is an important dimension in rehabilitation and physical activity, as it positively influences compliance to the exercise practice [32]. In rehabilitation, increment in self-efficacy has been linked to improved functionality and lowered pain among obese patients [33], and those with osteoarthritis [34]. Indeed, the contribution made by self-efficacy to physical function has to be considered in the management of subjects with joint pain or associated pathologies [35]. Given the close relationship between self-efficacy, motivation, and the actuation of a behavior, we can speculate that the increased self-efficacy influenced the attitude to continue exercising even at home.

The usability of the Playball®, evaluated through the SUS, showed an increment score of almost 10% at the end of the rehabilitation program. Although this result was not statistically significant, it underlined that the employment of the Playball® became progressively easier and more familiar over time. In addition, the SUS mean score at the end of the rehabilitation program exceeded the cut-off value of 68 reported in the scientific literature to define “good” the usability of a device [36]. Thus, though the system’s usability can be surely improved, no serious problems that limited its employment in the daily rehabilitation were registered. This scale has already proved to be a valid and reliable measure for assessing technological tools [37]. A study by Hägglund and colleagues recently employed the SUS to assess the Swedish patient accessible electronic health record [38]. A point in favor of the device usability is the high sensitivity of the pressure sensor, which allows the employment independently from the patients’ level of strength. Moreover, the games are designed to be performed without requiring a minimum range of motion.

Given the importance of therapeutic effectiveness, the purpose of digital therapies is also to be more attractive and entertaining than conventional treatments, allowing patients to be less focused on their health status (e.g., pain and discomfort) because of the more positive engagement during therapy. Indeed, two significant positive correlations were detected only in the PG. The Δ score from PACES significantly correlated with the Δ scores from the Self-efficacy dimension and the Intention to train at home dimension. Thus, our data suggested that the greater the enjoyment in rehabilitation with Playball®, the greater the self-efficacy and the stronger the intention to continue exercising at home once the rehabilitation is ended. This result highlighted that the digital therapy was positive and favored a greater patient engagement.

The present study has some potential limitations to acknowledge. First, though the short-term application of digital therapy could represent a novelty in this field, long-term compliance was not addressed in this study. Second, although researchers supervised the rehabilitation sessions, treatment fidelity was not objectively measured. Finally, results should be confirmed in a more significant number of patients since the sample size of the present study was not fully representative of the whole population suffering from shoulder impairments.

## Conclusions

Although digital therapy should not inevitably replace conventional methods, findings of the present study demonstrated that in shoulder rehabilitation, digital therapy with Playball® could be as effective as an equivalent non-digital therapy. Moreover, the device favored greater subject engagement, potentially leading to a greater predisposition to exercising individually at home after the supervised rehabilitation period. However, though the Playball® was globally evaluated as good in terms of usability, the non-significant result still encourages its improvement.

## Data Availability

All data generated or analyzed during this study are included in this published article.

## Abbreviations

CTRL: Control group
MVC: maximal voluntary contraction
PACES: Physical activity enjoyment scale
PG: Playball group
ROM: Range of motion
SUS: System Usability Scale
VAS-PAIN: Visual analogue scale for pain results
VAS-US: Visual analogue scale for usability results

## References

[1] Do Nascimento LMS, Bonfati LV, Freitas MLB, Mendes Junior JJA, Siqueira HV, Stevan SL. Sensors and systems for physical rehabilitation and health monitoring—a review. Sensors (Basel); 2020. pp. 1–28. 10.3390/s20154063 PMC7436073 32707749

[2] Wade SL, Narad ME, Shultz EL, Kurowski BG, Miley AE, Aguilar JM (2018). Technology-assisted rehabilitation interventions following pediatric brain injury. J Neurosurg Sci J Neurosurg Sci.

[3] Taylor MJD, McCormick D, Shawis T, Impson R, Griffin M. Activity-promoting gaming systems in exercise and rehabilitation. J Rehabil Res Dev. 2011. p. 1171–86. 10.1682/jrrd.2010.09.0171 22234662

[4] Geiger RA, Allen JB, O’Keefe J, Hicks RR (2001). Balance and mobility following stroke: effects of physical therapy interventions with and without biofeedback/forceplate training. Phys Ther.

[5] Walker C, Brouwer BJ, Culham EG (2000). Use of Visual Feedback in Retraining Balance following Acute Stroke. Phys Ther Oxford Academic.

[6] Decavele S, Ortibus E, Van Campenhout A, Molenaers G, Jansen B, Omelina L (2020). The Effect of a Rehabilitation Specific Gaming Software platform to Achieve Individual Physiotherapy Goals in children with severe spastic cerebral palsy: a randomized crossover trial. Games Health J Games Health J.

[7] De Kloet AJ, Berger MAM, Verhoeven IMAJ, Van Stein Callenfels K, Vlieland TPMV (2012). Gaming supports youth with acquired brain injury? A pilot study. Brain Inj Brain Inj.

[8] Bethi SR, Rajkumar A, Vulpi F, Raghavan P, Kapila V. Wearable Inertial Sensors for Exergames and Rehabilitation. Proc Annu Int conf IEEE Eng Med Biol soc EMBS. Volume 2020–July. Institute of Electrical and Electronics Engineers Inc.; 2020. pp. 4579–82. 10.1109/EMBC44109.2020.9175428 33019013

[9] Dennett AM, Taylor NF (2015). Machines that go “ping” may improve balance but may not improve mobility or reduce risk of falls: a systematic review. J Rehabil Med J Rehabil Med.

[10] Lohse K, Shirzad N, Verster A, Hodges N, Van Der Loos HFM (2013). Video games and rehabilitation: using design principles to enhance engagement in physical therapy. J Neurol Phys Ther J Neurol Phys Ther.

[11] Kwon JS, Park MJ, Yoon IJ, Park SH (2012). Effects of virtual reality on upper extremity function and activities of daily living performance in acute stroke: a double-blind randomized clinical trial. NeuroRehabilitation NeuroRehabilitation.

[12] Subramanian S, Dahl Y, Skjæret Maroni N, Vereijken B, Svanæs D (2020). Assessing motivational differences between Young and older adults when playing an Exergame. Games Health J Games Health J.

[13] Domínguez-Téllez P, Moral-Muñoz JA, Salazar A, Casado-Fernández E, Lucena-Antón D (2020). Game-based virtual reality interventions to improve Upper Limb Motor function and quality of Life after Stroke: systematic review and Meta-analysis. Games Health J Games Health J.

[14] McNulty PA (2012). Games for Rehabilitation: Wii-based Movement Therapy improves Poststroke Movement ability. Games Health J Games Health J.

[15] Putrino D, Zanders H, Hamilton T, Rykman A, Lee P, Edwards DJ (2017). Patient Engagement is related to impairment reduction during Digital Game-Based therapy in stroke. Games Health J Games Health J.

[16] Hawker GA, Mian S, Kendzerska T, French M. Measures of adult pain: Visual Analog Scale for Pain (VAS Pain), Numeric Rating Scale for Pain (NRS Pain), McGill Pain Questionnaire (MPQ), Short-Form McGill Pain Questionnaire (SF-MPQ), Chronic Pain Grade Scale (CPGS), Short Form-36 Bodily Pain Scale (SF. Arthritis Care Res (Hoboken). Arthritis Care Res (Hoboken); 2011;63 Suppl 1. 10.1002/acr.20543 22588748

[17] Leggin BG, Michener LA, Shaffer MA, Brenneman SK, Iannotti JP, Williams GR (2006). The Penn shoulder score: reliability and validity. J Orthop Sports Phys Ther J Orthop Sports Phys Ther.

[18] Carraro A, Elliot CA, Gobbi E (2019). Perceived treadmill function is correlated with enjoyment of use in trained runners: a user-centred approach. Appl Ergon Appl Ergon.

[19] Carraro A, Gobbi E, Ferri I, Benvenuti P, Zanuso S (2014). Enjoyment perception during exercise with aerobic machines. Percept Mot Skills Percept Mot Skills.

[20] Bandura A, Freeman WH, Lightsey R, Self-Efficacy. The Exercise of Control. J Cogn Psychother. Springer; 1999;13:158–66. Available from: https://connect.springerpub.com/content.

[21] Ajzen I. From Intentions to Actions: A Theory of Planned Behavior. Action Control [Internet]. Springer, Berlin, Heidelberg; 1985 [cited 2022 Feb 4];11–39. Available from: https://link.springer.com/chapter/10.1007/978-3-642-69746-3_2.

[22] Ajzen I, Perceived Behavioral, Control. Self-Efficacy, Locus of Control, and the Theory of Planned Behavior1. J Appl Soc Psychol. John Wiley & Sons, Ltd; 2002 [cited 2022 Feb 4];32:665–83. Available from: https://onlinelibrary.wiley.com/doi/full/10.1111/j.1559-1816.2002.tb00236.x.

[23] Borsci S, Federici S, Lauriola M. On the dimensionality of the System Usability Scale: A test of alternative measurement models. Cogn Process. Springer; 2009;10:193–7. Available from: https://link.springer.com/article/10.1007/s10339-009-0268-9. 10.1007/s10339-009-0268-9 19565283

[24] Brooke J, Jordan P, Thomas B, Weerdmeester B, McClelland I (1996). SUS: a “quick and dirty” usability scale. Usability Eval Ind.

[25] Santos P, Machado T, Santos L, Ribeiro N, Melo A (2019). Efficacy of the Nintendo Wii combination with Conventional exercises in the rehabilitation of individuals with Parkinson’s disease: a randomized clinical trial. NeuroRehabilitation IOS Press.

[26] Sin H, Lee G (2013). Additional virtual reality training using Xbox kinect in stroke survivors with hemiplegia. Am J Phys Med Rehabil.

[27] Rizzo JR, Thai P, Li EJ, Tung T, Hudson TE, Herrera J (2017). Structured Wii protocol for rehabilitation of shoulder impingement syndrome: a pilot study. Ann Phys Rehabil Med Elsevier Masson.

[28] Tsutsumi T, Don BM, Zaichkowsky LD, Delizonna LL (1997). Physical fitness and psychological benefits of strength training in community dwelling older adults. Appl Hum Sci Appl Hum Sci.

[29] Dobkin BH (2017). A Rehabilitation-Internet-of-things in the home to Augment Motor Skills and Exercise Training. Neurorehabil neural repair. Neurorehabil Neural Repair.

[30] Vealey R, Chase M. Self-confidence in sport. In: Horn T, editor. Adv Sport Psychol. Human Kinetics; 2008. pp. 68–97.

[31] Bandura A (1986). Social foundations of thought and action: a social cognitive theory. Englewood Cliffs, editor.

[32] Mcauley E, Szabo A, Gothe N, Olson EA (2011). Self-efficacy: implications for physical activity, function, and functional Limitations in older adults. Am J Lifestyle Med Am J Lifestyle Med.

[33] Mihalko SL, Cox P, Beavers DP, Miller GD, Nicklas BJ, Lyles M (2019). Effect of intensive diet and exercise on self-efficacy in overweight and obese adults with knee osteoarthritis: the IDEA randomized clinical trial. Transl Behav Med Oxford Academic.

[34] Baker KR, Nelson ME, Felson DT, Layne JE, Sarno R, Roubenoff R. The efficacy of home based progressive strength training in older adults with knee osteoarthritis: a randomized controlled trial. J Rheumatol. 2001;28. 11469475

[35] Hermsen LAH, Van Der Wouden JC, Leone SS, Smalbrugge M, Van Der Horst HE, Dekker J. The longitudinal association of cognitive appraisals and coping strategies with physical functioning in older adults with joint pain and comorbidity: a cohort study. BMC Geriatr BMC Geriatr; 2016;16. 10.1186/s12877-016-0204-7 PMC4730621 26818402

[36] Brooke J (2013). SUS: a retrospective Display design for fault diagnosis view project SUS. A Retrospective.

[37] Friesen E (2017). Measuring AT usability with the Modified System Usability Scale (SUS). Stud Health Technol Inform.

[38] Hägglund M, Scandurra I. User evaluation of the swedish patient Accessible Electronic Health Record: System Usability Scale. JMIR hum factors. Volume 8. JMIR Hum Factors; 2021. 10.2196/24927 PMC8367176 34313596

