# Correction: Effectiveness and therapeutic compliance of digital therapy in shoulder rehabilitation: a randomized controlled trial

**DOI:** 10.1186/s12984-024-01314-z

**Published:** 2024-02-07

**Authors:** Alex Rizzato, Martina Pizzichemi, Erica Gobbi, Adriana Gerardi, Claudia Fortin, Ancuta Copcia, Antonio Paoli, Giuseppe Marcolin

**Affiliations:** 1 Department of Biomedical Sciences, University of Padova, Via Marzolo, 3, 35131 Padova, Italy; 2 School of Human Movement Sciences, University of Padova, Padova, Italy; 3 Department of Biomolecular Sciences, University of Urbino Carlo Bo, Urbino, Italy; 4 Data Medica Group, Synlab S.P.A, CEMES, Padova, Italy

**Correction: Journal of NeuroEngineering and Rehabilitation (2023) 20:87** 10.1186/s12984-023-01188-7

Following publication of the original article [1], the alignment and the value of the Table 3 has been corrected as shown below:

**Table 3:** Results of the MANOVA analysis for the engagement variables. Post-hoc comparisons show the significant main effect of time (T0 vs. T1) for both control (CTRL) and PlayBall (PG) groups. Significantly different from PRE (T0): * (p < 0.05)

	CTRL		PG	
	PRE (T_0_)	POST (T_1_)	PRE (T_0_)	POST (T_1_)
PACES	53.90 ± 4.52	51.63 ± 10.97	50.18 ± 9.44	51.63 ± 8.41
Self-efficacy	5.36 ± 1.20	6.18 ± 0.87*	5.0 0± 1.09	5.18 ± 1.07*
Attitude to train at home	5.67 ± 1.59	6.29 ± 0.66*	4.67 ± 1.24	5.48 ± 1.41*
Intention to train at home	6.09 ± 1.62	5.90 ± 1.85	5.50 ± 1.56	6.18 ± 1.07

The original article has been corrected.
